# De La Lithotripsie—*Par* Leroy D’Etiolle, Docteur En Médecine

**Published:** 1838-03-28

**Authors:** 


De la Lithotripsie—*Par* Leroy d'Etiolle, Docteur en Médecine. *Mémoire, No.* 1. *Paris*, 1837.

This work, as will be seen by the author's preface, "is the first part of a series of memoirs which he proposes to publish on the diseases of the urinary passages." It contains upwards of 320 pages; and is copiously supplied with *wood-cuts*, picturing the various instruments which have been progressively devised and employed in Lithotripsy; and the text explaining the uses and advantages of, and objections to them. The author's opinions, views, and practice, are lucidly and scientifically explained, and enforced by the appropriate detail of cases. Many of them are exceedingly interesting and instructive. Appended to this memoir are two historical tables of Lithotripsy, explanatory of the instruments, giving the inventor's name; when introduced; and furnishing also references to published accounts of them, &c.

The work is not yet within the reach of the American faculty generally; but when attainable, it is worthy the attentive examination even of those who are sceptical on the subject of the present or prospective value of this modern innovation in Surgery as *an extinguisher* of all other modes of removing urinary calculi. We have the pleasure of a personal acquaintance with the author of this work; have enjoyed many conversations with him on the subject of which he treats; have witnessed his hospital practice; and have accompanied him to the bedside of his respectable private patients; and do not hesitate to express our belief that he is one of the most enlightened, judicious, and experienced lithotrite surgeons in Europe; and as an operator, superior to most and inferior to none in his immediate field of practice—the city of Paris. We advise the American students who may visit the French Schools, to profit by a course of lectures delivered by this gentleman, during the winter season, on this, his favorite subject. J. R. B.

